# Adenosine Stimulate Proliferation and Migration in Triple Negative Breast Cancer Cells

**DOI:** 10.1371/journal.pone.0167445

**Published:** 2016-12-02

**Authors:** Miriam Fernandez-Gallardo, Ricardo González-Ramírez, Alejandro Sandoval, Ricardo Felix, Eduardo Monjaraz

**Affiliations:** 1 Laboratorio de Neuroendocrinología Molecular, Instituto de Fisiología, Benemérita Universidad Autónoma de Puebla (BUAP), Puebla, México; 2 Departmento de Biología Molecular e Histocompatibilidad, Hospital General "Dr. Manuel Gea González", Secretaría de Salud, Ciudad de México, México; 3 Facultad de Estudios Superiores Iztacala, Universidad Nacional Autónoma de México (UNAM), Tlalnepantla, México; 4 Departmento de Biología Celular, Centro de Investigación y de Estudios Avanzados del Instituto Politécnico Nacional (Cinvestav-IPN), Ciudad de México, México; University of South Alabama Mitchell Cancer Institute, UNITED STATES

## Abstract

Emerging evidence suggests that the adenosine (Ado) receptors may play crucial roles in tumor progression. Here, we show that Ado increases proliferation and migration in a triple negative breast cancer model, the MDA-MB 231 cell line. The use of specific agonists and antagonists evidenced that these effects depend on the activation of the A_2B_ receptor, which then triggers an intracellular response mediated by the adenylate cyclase/PKA/cAMP signaling pathway. Ado also increases the expression of Na_V_1.5 channels, a potential biomarker in breast cancer. Together, these data suggest important roles of the A_2B_ receptors and Na_V_1.5 channels in the Ado-induced increase in proliferation and migration of the MDA-MB 231 cells.

## Introduction

Development and progression of cancer depend on a variety of chemical messengers including growth factors, cytokines and molecules such as adenosine-5′-triphosphate (ATP) and adenosine (Ado), among many others. It is also acknowledged that tumor hypoxia increases cell metabolic rate with a high demand for ATP, which is metabolized to Ado that promotes angiogenesis and induces an inflammatory reaction, two hallmark features of tumor growth [1]. In response to hypoxia, cells synthesize and express the hypoxia-inducible factor 1α (HIF-1α) a transcription factor that controls the expression of diverse genes associated with cell metabolism and proliferation such as the vascular endothelial growth factor (VEGF) and the basic fibroblast growth factor (bFGF) that act as angiogenic factors [2],[3],[4],[5].

Interestingly, Ado also stimulates VEGF expression leading to increased intratumoral blood flow and cell proliferation by acting on purinergic P1 receptors, a family of four G protein-coupled receptors named A_1_, A_2A_, A_2B_ and A_3_ [6],[7],[8]. These receptors differ in their affinity for Ado and the signaling pathway activated in the target cell. Ado binds A_1_, A_2A_ and A_3_ with high affinity and shows low affinity for A_2B_ receptors [9]. A_1_ is coupled to different members of the G protein family G_i/o_ while A_3_ is coupled to G_i_ and G_q_ proteins. Both receptors inhibit adenylate cyclase (AC), activate phospholipase C (PLC) and reduce Ca^2+^ conductance [10]. In contrast, activation of A_2A_ and A_2B_ coupled to G_s_/G_olf_ and G_s_/G_q_ proteins, respectively, increases AC and PLC activity, and causes an inositol-1,4,5-tris-phosphate (IP_3_)-dependent increase in intracellular Ca^2+^ [10],[11].

Ado has also shown to interfere with the recognition of tumor cells by the immune system exerting an immunosuppressive effect [12],[13],[14]. In addition, RT-PCR and *in situ* hybridization assays have revealed the presence of A_2B_ in many cell types and tissues, and that is activated only in the presence of high Ado concentrations, which occurs in some pathological conditions [15]. Likewise, A_2B_ activation in the microvasculature modulates the expression of angiogenic factors VEGF, bFGF, and IL-8 [11] and the proliferation of endothelial cells, which may have an impact on tumor growth and invasiveness by inducing neovascularization in the area surrounding the tumor [16].

Breast cancer affects over one million patients every year. Recent progresses in the biology of this disease have led to improved patient survival. There remains however, a subgroup of patients called “triple-negative” characterized by the lack of expression of receptors to estrogen, progesterone and human epidermal growth factor receptor 2 (HERB-2) for whom treatment offers only limited benefits. Although the triple-negative subtype represent a relatively small number of cases of breast cancer, its study becomes very relevant given the significant number of deaths associated with it. In addition, there has been less progress in the treatment of the triple negative than in other subtypes breast cancer [17]. For these reasons, it is imperative to find new alternatives for the diagnosis and treatment of triple negative breast cancer.

In this study we aimed to investigate the molecular mechanism by which Ado stimulates cell proliferation in the tumor cell line MDA-MB 231 derived from human breast cancer at late-stages, where the expression of A_2B_ is increased [18]. This cell line is a prominent system for studying the triple-negative breast cancer.

## Materials and Methods

### Cell culture

MDA-MB-231 human breast cancer cell line, a generous gift of Dr. E. Pérez-Salazar (Cinvestav, Mexico), were maintained in Advanced RPMI-1640 culture medium supplemented with 100 U ml^–1^ penicillin, 100 mg ml^–1^ streptomycin and 3% fetal bovine serum (FBS) at a constant temperature of 37°C with a humidified atmosphere of 5% CO_2_. The cells were passaged once a week. For growth experiments, cells were trypsinized and 100 μl of the cell suspension were plated in 100^2^-mm diameter wells in 10 ml of the growth medium. The cells were cultured for 48–72 h before treatment.

### Cell proliferation

Proliferation was assessed by incorporation of [^3^H]-thymidine into DNA strands during the S phase of the cell cycle. Briefly, cells were seeded in multiwell dishes and treated with Ado at different concentrations for 48–72 h. In parallel, cells were also grown in the presence of cAMP-PKA signaling modulators. Cells were then incubated for 4 h with 0.01 μCi/well [methyl,1’,2’-^3^H]-thymidine in serum-free medium, rinsed twice with cold phosphate-buffered saline (PBS), prefixed for 3 min with a formulation of 1:1 PBS/fixative (70% ethanol/30% acetic acid). After washing, cells were then fixed in ethanol for 10 min at 37°C and washed for 10 min with SDS (1%). The fixed cells were incubated for 20 min in 100 μl of a 0.25 M NaOH before an equal volume of distilled water was added. The hydrolysate was then transferred to scintillation vials with 1.2 ml of scintillation fluid, and samples were counted for 5 min in a β-counter.

### Transwell migration assay

The migration assay was performed in 24-well transwell chambers with 8 μm polycarbonate Nucleopore filters. Initially, the filters were washed with serum-free DMEM, and placed into 24-well plates. The lower chambers contained Advanced DMEM with 3% FBS. For the upper chambers, 3×10^4^ cells were resuspended in 200 μl Advanced DMEM FBS free medium. Plates were then incubated at 37°C in 5% CO_2_. After 12 h, the non-migrating cells in the chambers were removed, while the cells that migrated through the membranes were fixed with methanol and stained with 0.5% crystal violet. The number of migrating cells was quantified by counting ten randomly selected visual fields.

### Cell counts using the Scepter

MDA-MB 231 cells were trypsinized and harvested during the exponential growth phase and a total of 5×10^5^ cells were plated into 6-well culture plates (2 ml/well). After 24 h, the cells were exposed to various concentrations of Adenosine for the indicated time periods. The Scepter cell counter (Millipore) was used to count cell numbers. Counts from triplicate wells were averaged.

### Reverse transcription-polymerase chain reaction (RT-PCR)

Total RNA was extracted from MDA-MB 231cells using the ZR RNA MiniPrep kit. For cDNA synthesis, total RNA samples (2 μg) were subjected to reverse transcription with 0.8 μl random primers and 1 μl MuLV enzyme in 20 μl of reaction mixture at 37°C for 2 h. cDNA amplification was carried out by PCR in a total volume of 20 μl: 1.5 μl of cDNA, 10 μl of PyroStartTM Fast PCR Master Mix, 1 μl of each primer and 6.5 μl H_2_O. PCR primers used are given in S1 Table. The reactions were performed as follows: 30 cycles of 95°C for 45 s, 55°C for 30 s and 72°C for 1 min. PCR products were electrophoresed on 1.2% agarose gels and stained with ethidium bromide. The identity of the amplicons was confirmed by automated sequencing.

### RNA extraction and quantitative PCR

Total RNA was extracted using TriPure Isolation Reagent. One ml of reagent was added to the culture dish, homogenized and incubated at room temperature for 5 min. After chloroform extraction and precipitation with isopropanol, RNA was washed, dissolved in RNase-free water and analyzed in an agarose gel. Quantitative PCR was performed on a CFX96 Real-Time PCR Detection System. Reactions were performed in triplicate in a volume of 20 μL of a mixture containing 1X Power SYBR Green RNA-to-CT 1-Step Kit, 200 nM of Ado-receptor primers (S2 Table), 1X RT Enzyme Mix and 30 ng of total RNA. Cycling conditions were: reverse transcription 30 min at 48°C, activation of AmpliTaq Gold DNA Polymerase 10 min at 95°C, followed by 40 cycles of denaturation for 15 s at 95°C, and annealing-extension at 60°C for 30 s. Amplification results were analyzed using CFX Manager software.

### Immunohistochemistry

Cells were fixed in 2% glutaraldehyde in PBS for 30 min and washed in 4.5% fructose and incubated in H_2_O_2_ to block endogenous peroxidase followed by 3 h incubation in horse serum to block nonspecific binding. Cells were then incubated in the absence or the presence of primary anti-A_2B_ receptor (H-40; Santa Cruz Biotechnology), at 1:300 dilution, overnight at 4°C followed by the appropriate biotinylated secondary antibody for 2 h at room temperature (1:500 dilution). The antibody was localized using streptavidin-HRP for 20 min at room temperature.

### cAMP assay

MDA-MB 231 cells were grown for 48 h in 24-well dishes in glucose-containing modified Krebs Henseleit (KH) medium (in mM): 116 NaCl, 3 KCl, 1 MgSO_4_, 1.2 KH_2_PO_4_, 25 NaHCO_3_, 1 CaCl_2_, pH 7.4). Cells were washed in KH, suspended in fresh medium, and kept at 37°C for 15 min in the presence of IBMX (1 mM). Then, the KH was changed and [^3^H]-adenine was added to the incubation medium. After 60 min at 37°C, Ado at different concentrations was added. The reaction was terminated with 550 μl of 10% trichloroacetic acid, the mixture was homogenized and centrifuged, and the supernatants (of which 50 μl aliquots were transferred into scintillation vials for the determination of total activity) were decanted into test tubes. The [^3^H]-cAMP formed was isolated by sequential chromatography (Dowex 50Wx4 and aluminum oxide columns). The final eluate was tested for radioactivity in a liquid scintillation counter.

### Elaectrophysiology

Sodium currents in MDA-MB-231 cells were recorded according to the whole cell configuration of the patch clamp technique as described previously [19] in a bath solution containing (mM): 140 NaCl; 5 KCl; 2 CaCl_2_; 10 HEPES; 10 glucose (~300 mOsm/L; pH 7.4). Patch pipettes were filled with a solution containing 105 CsCl; 35 NaCl; 1 MgCl_2_; 1 EGTA; 10 HEPES; 4 Mg-ATP; 0.1 GTP (~290 mOsm/L; pH 7.4). Recordings were made using an Axopatch 200B amplifier. Data acquisition and analysis were performed using pClamp10 software and Sigma Plot 11.0. Linear leak and parasitic capacitance components were subtracted on-line using a standard P/4 protocol. Membrane capacitance (*C*m) was used to normalize currents. Patch pipettes were made from borosilicate glass and the typical electrical resistance was 2–3 MΩ when filled with the internal solution. Currents were evoked by 50 ms depolarizing voltage steps ranging from -70 to +60 mV in 10 mV increments from a holding potential of -80 mV.

## Results

**Fig 1:**
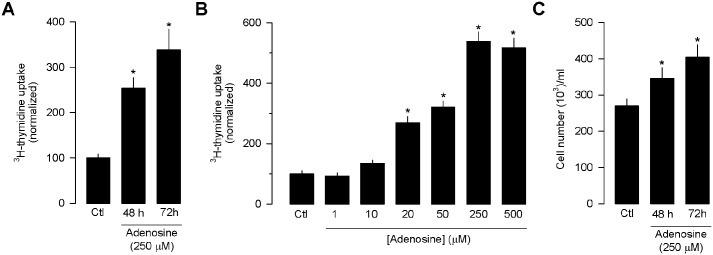
Effect of Ado on proliferation of MDA-MB-231 cells. (A) Incorporation of [^3^H]-thymidine into cells incubated for 48–72 h in the absence (Control) or the presence of Ado (50 μM). (B) Dose dependence of the effects of Ado on the incorporation of [^3^H]-thymidine. Cells were cultured for 48–72 h in the absence (Control) or presence of various concentrations of Ado. (C) Effect of Ado on cell growth. Cells were plated and after 48or 72 h of incubation, attached cells were trypsinized and counted with the aid of a using a handheld automated cell counter (Scepter 2.0, Millipore).

To study the effects of Ado on the proliferation of the hormone-independent (triple-negative) MDA-MB-231 human breast cancer cell line, we first analyzed by the [^3^H]-thymidine incorporation in serum-starved cells. After 48–72 h of treatment, Ado (250 μM) caused a 2-3-fold increase in [^3^H]-thymidine incorporation (Fig 1A). This effect was dose dependent; the stimulation of DNA synthesis by Ado, which was detectable at a concentration of 20 μM, reached a maximum at 250 μM and was sustained up to 500 μM, the highest concentration tested (Fig 1B). To better assess the stimulatory effect of Ado, MDA-MB-231 cells were incubated after a 1-d period of serum starvation in the absence or the presence of 250 μM Ado, and the attached cells were counted after 48–72 h. The results of this analysis showed that the number of Ado-treated cells increased 20–35% (Fig 1C).

**Fig 2:**
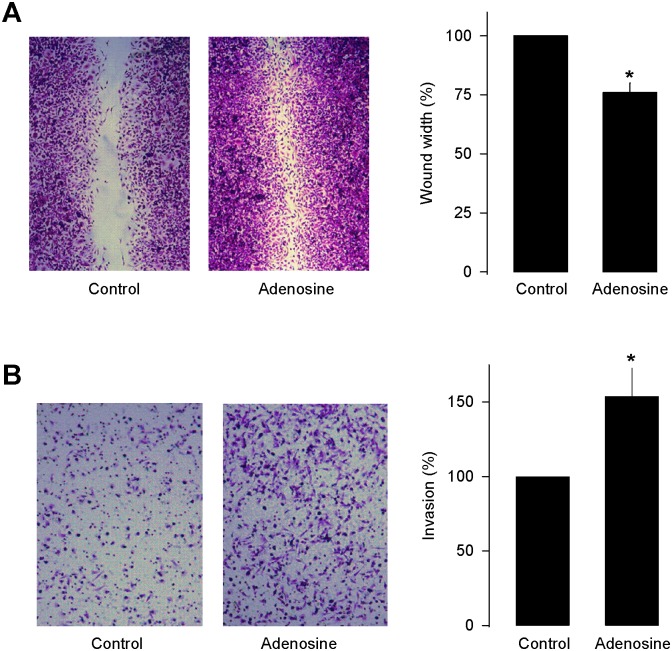
Effect of Ado on migration of MDA-MB-231 cells. (A) Wound-healing migration assay for MDA-MB-231 cells in the absence and presence of 50 μM Ado (left panel). The percentage of migration in the transwell assay is shown in right panel. (B) The cell migration was assessed by the transwell migration assay in MDA-MB-231 cells incubated in the absence (control) or presence of 50 μM Ado. Shown are representative micrographs of the migrated cells stained with crystal violet (left panel) and the percentage of migrated cell (right panel). The results represent the means ± SE of three independent experiments performed in triplicate. Asterisks denote significant differences (p<0.05) compared to control.

To examine whether the treatment with Ado (50 μM) also conferred migratory properties to the MDA-MB-231 cells, a wounding assay in which cells are induced to migrate into a wound created by scratching confluent cultures with a pipette tip was used. As can be seen in Fig 2A, in contrast to untreated cells which migrated poorly, Ado-treated cells significantly invaded the wound. Likewise, as an alternative for measuring cell motility, transwell-migration assays were performed. In these experiments treatment with Ado (50 μM) resulted in an enhanced migratory response of MDA-MB-231 cells (Fig 2B).

**Fig 3:**
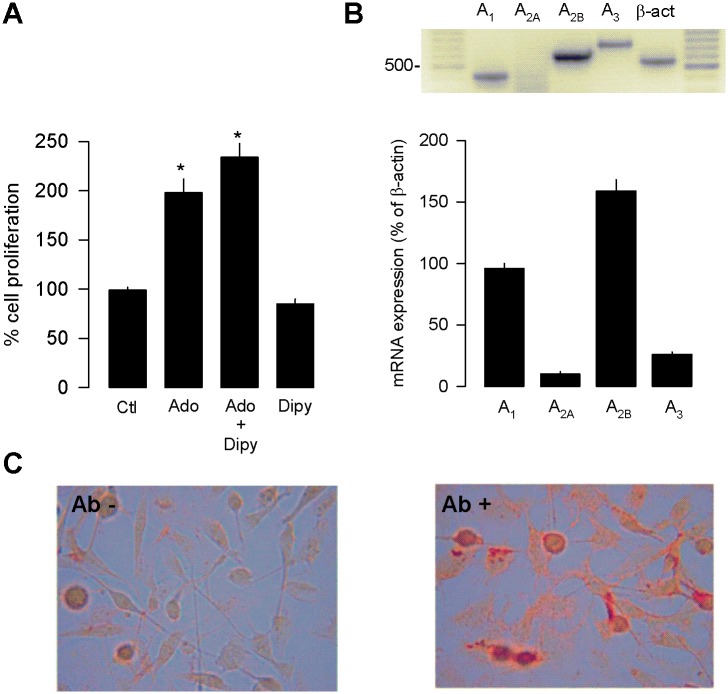
Expression of Ado receptors in MDA-MB-231 cells. (A). Percentage of MDA-MB-231 cell proliferation in the presence of Ado with or without dipyridamole (Dipy) an inhibitor of nucleoside transport. (B) RT-PCR of Ado receptor mRNAs in MDA-MB-231 cells. A representative gel is shown (upper panel). Levels of Ado receptor mRNAs were estimated from gene quantitative RT-PCR data and expressed as a percentage of β-actin mRNA expression (lower panel). Cells were incubated for 72 h in the absence (Control) or presence of Ado (50 μM), or the A_2B_ receptor agonist Bay 60–6583 (1 μM). (C) Immunocytochemistry for the A_2B_ receptor was performed to verify expression at the protein level in the absence (Ab -) or the presence of specific antibodies (Ab +).

Given that Ado in addition to interact with cell surface receptors can be also recycled through dipyridamole-sensitive carriers [20], we next measured the [^3^H]-thymidine incorporation in MDA-MB-231 cells in the absence or presence of the inhibitor of Ado reuptake dipyridamole. The use of the inhibitor did not prevent the stimulatory effect of Ado on MDA-MB-231 cell proliferation (Fig 3A). It is therefore likely that the effects of Ado on [^3^H]-thymidine incorporation in MDA-MB-231 cells are mediated by activation of receptors in the cell surface. Consequently, next we sought to determine the expression profile of the mRNAs encoding the Ado-receptors in the MDA-MB-231 cells. To this end, primers for Ado receptors were designed, and the level of expression of the corresponding transcripts was assessed by RT-PCR (S1 and S2 Tables). Total RNA was extracted from MDA-MB-231 cells and optimal conditions for quantitative RT-PCR were then used to amplify the cDNAs for the Ado receptors. The results in Fig 3B show that all Ado receptor mRNAs were expressed in the MDA-MB-231 cells, though their level of expression was different. To quantify these differences, the receptor signals were expressed as a percentage of β-actin expression. The results revealed that both A_2A_ and A_3_ receptors are less abundant than β-actin in the MDA-MB-231 cells, while the relative expression of the A_1_ receptor is similar to actin expression. In contrast, the intensity of the A_2B_ receptor mRNA signal was significantly higher in comparison to the β-actin signal. When compared among them, the quantitative analysis showed that the level of expression of the A_2B_ receptor was ~3.5-, 7- and 4-fold higher than the level of expression of the A_1_, A_2A_ and A_3_ receptors, respectively.

Panjehpour and co-workers (2005) showed that the incubation of MDA-MB-231 cells in the presence of non-selective Ado receptor agonists (NECA and PHPNECA) stimulated adenylate cyclase activity and increased the intracellular Ca^2+^ levels in a phospholipase C-dependent manner [21]. Interestingly, these responses were not present when the cells were incubated in the presence of agonists selective for A_1_, A_2A_ and A_3_ Ado receptors, and consequently the authors suggested that they were mediated by the activation of A_2B_ receptors. However, a direct experimental test of this hypothesis was not provided. In the present report, by using immunocytochemistry we first examined the expression of the A_2B_ receptor. The results indicated a negative staining for the A_2B_ receptor in MDA-MB-231 cells in the control condition, in which the primary antibody was omitted, whereas a substantial amount of analyzed cells incubated with the anti-A_2B_ receptor antibody expressed the protein of interest (Fig 3C).

**Fig 4:**
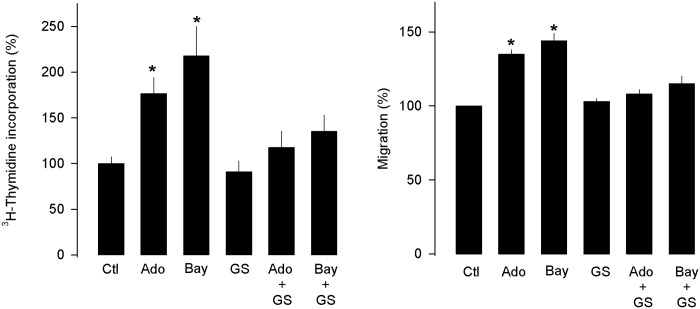
Ado increases proliferation in MDA-MB-231 cells via A_2B_ receptor activation. The activation of the A_2B_ receptor increases [^3^H]-thymidine incorporation (A) and cell migration expressed as a percentage of the control (B). In contrast, incubation for 72 h in the presence of an A_2B_ receptor antagonist (GS6201; 100 nM) prevented the Ado- and Bay 60-6583-mediated enhancement of [^3^H]-thymidine incorporation and migration in MDA-MB-231 cells.

Next, we examined the role of the A_2B_ receptor activation in the Ado-mediated responses of the MDA-MB-231 cells. This was accomplished by using an agonist (Bay 60–6583) and an antagonist (GS-6201) of the A_2B_ receptor. After 72 h of treatment, activation of the A_2B_ receptor with Ado (50 μM) caused a significant ~2-fold increase in [^3^H]-thymidine incorporation in the MDA-MB-231 cells (Fig 4B). Similar results were observed with the use of Bay 60–6583. In sharp contrast, the use of the A_2B_ receptor antagonist (GS-6201) considerably prevented both the Ado- and Bay 60-6583-mediated increase in [^3^H]-thymidine incorporation. These results suggest that Ado increases proliferation in the MDA-MB-231 cells via the A_2B_ receptor activation. Similar results were observed when the migratory response of the MDA-MB-231 cells was assessed (Fig 4B).

**Fig 5:**
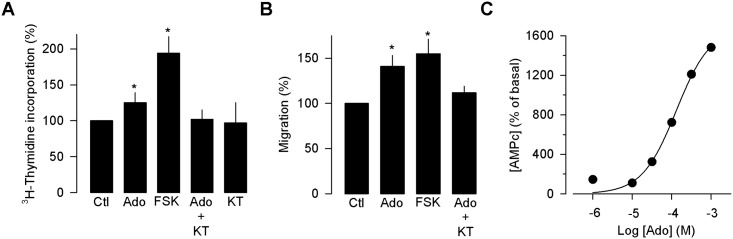
The adenylate cyclase/PKA pathway is involved in the effects of Ado in MDA-MB-231 cells. Incubation of MDA-MB-231 cells in the presence of an adenylate cyclase agonist (FSK; 10 μM) mimics the Ado-induced effect on cell proliferation (A) and migration (B). In contrast, incubation in the presence of the antagonist KT-5720 (500 nM) prevented the effect of Ado on MDA-MB-231 cell proliferation and migration. (C) The activation of Ado receptors in MDA-MB-231 cells causes a concentration-dependent increase of [^3^H]-cAMP.

In order to identify the signaling cascade underlying the A_2B_ receptor-mediated cell proliferation response, possible pathways were probed. First, to assess a potential role of adenylyl cyclase/protein kinase A (PKA) signaling pathway in the action of Ado, the effect of a specific activator of adenylyl cyclase forskolin (FSK) was tested. In addition, an antagonist of this enzyme (KT-5720), was also used. The results showed that the use of FSK mimics the Ado-induced increase in proliferation and migration of the MDA-MB 231 cells (Fig 5A and 5B), and KT-5720 was able to prevent the effects of Ado treatment in the MDA-MB-231 cells. These results suggest an important role of adenylyl cyclase/PKA signaling pathway in the proliferative and invasive effects caused by Ado. To investigate this in more detail, the synthesis of [^3^H]-cAMP was measured in extracts of MDA-MB-231 cells incubated 48–72 h in the presence of increasing concentrations of Ado. As expected from the results obtained with FSK and KT-5720, Ado was able of inducing cAMP synthesis in the μM range. Fifty percent of the maximum effect on cAMP synthesis was attained at the concentration of 100 μM (Fig 5C), supporting the hypothesis that adenylyl cyclase and PKA may be implicated in the mechanism of action of Ado on the MDA-MB-231 cell line proliferation and migration via the A_2B_ receptor activation.

**Fig 6:**
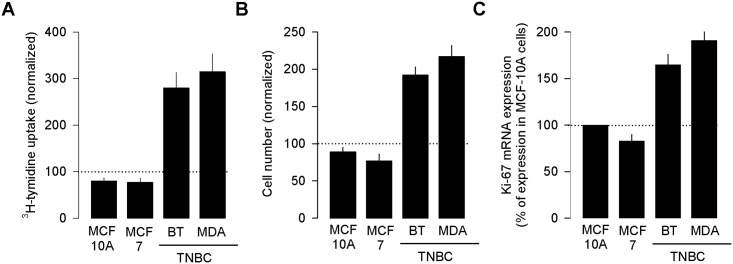
Adenosine increases proliferation in triple negative breast cancer cells (TNBC) cells. Effects of Ado on [^3^H]-thymidine incorporation (A) and cell number (B) in TNBC (MDA-MB-231 and BT-549) and non-TNBC (MCF-10A and MCF-7) cell lines. Cells were treated for 3 days with 50 μM of Ado. (C) Comparison of the effects of Ado on Ki-67 mRNA expression in breast cancer cell lines as A and B. Cells were treated with 50 μM of Ado for 3 days. Percentages of [^3^H]-thymidine incorporation, cell number and Ki-67 mRNA expression in treated cells (bars) are compared with that in control cells (dotted lines).

In order to investigate whether this effect is not specific for only one TNBC cell line (MDA-MB 231), a repeat of these studies in other triple negative breast cancer cell line (BT-549) was performed. In addition, the use of two non-TNBC cell lines was included also to examine whether the effect was specific to TNBC cells lines. The results of these experiments are summarized in Fig 6. As can be seen, after 48–72 h of treatment, Ado (250 μM) caused a ~3-fold increase in [^3^H]-thymidine incorporation in the two TNBC cell lines (Fig 6A). Conversely, the treatment with Ado had no appreciable effects on proliferation of the two non-TNBC cell lines investigated (MCF-10A and MCF-7). Consistent with this, the number of Ado-treated cells in TNBC cell lines showed a ~2-fold increase with respect to the untreated cultures, and as expected from the results of the [^3^H]-thymidine incorporation assays, the number of non-TNBC cells remained unchanged even when such cultures were treated with Ado (Fig 6B). Likewise, the mRNA expression of Ki-67, a proliferation-related nuclear antigen, was also determined in the TNBC and non-TNBC cell lines in presence and absence of Ado. Semi-quantitative analysis of RT-PCR experiments showed a significant increase (>75%) in Ki-67 mRNA expression after Ado treatment only in the two TNBC cell lines compared with untreated controls (Fig 6C).

**Fig 7:**
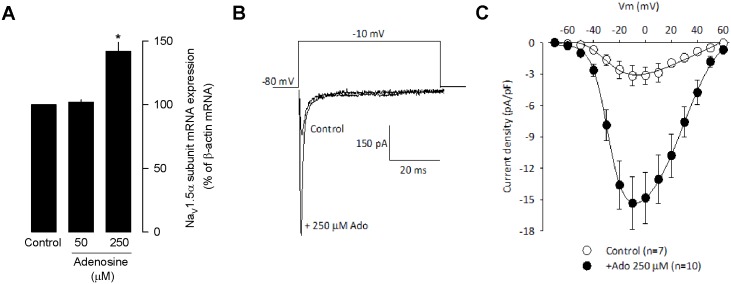
Ado increases Na_V_1.5 channel functional expression. (A) Relative effect of Ado on Na_V_1.5 mRNA expression in the MDA-MB-231 cells as compared to control. (B) Representative superimposed trace currents through Na_V_ channels in MDA-MB-231 cells in the absence and the presence of Ado (250 μM). The currents were generated by pulsing the membrane potential from a holding voltage of -80 mV to 0 mV for 50 ms. (C) Mean current-voltage relationship obtained from MDA-MB-231 control cells and cells incubated 48 h in the presence of Ado (250 μM). Cells were depolarized from -70 to +60 mV for 50 ms by 10-mV increments from a holding potential of -80 mV.

**Fig 8:**
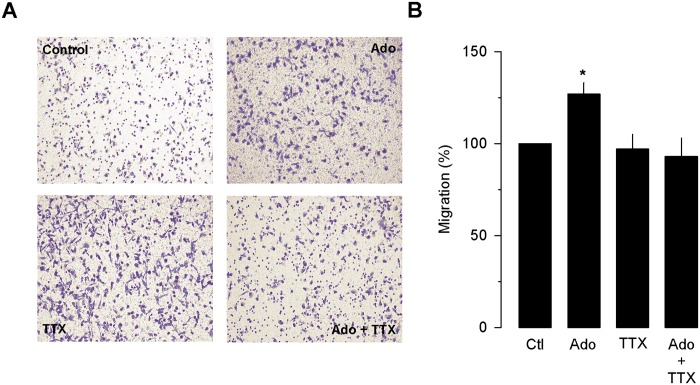
Effect of tetrodotoxin (TTX) on migration of MDA-MB-231 cells. The cell migration was assessed by the transwell migration assay in MDA-MB-231 cells incubated in the absence (control) or presence of 50 μM Ado, as well as 10 μM TTX as indicated. Shown are representative micrographs of the migrated cells stained with crystal violet (A) and the percentage of migrating cells is summarized in the bar chart (B).

Last, in the MDA-MB-231 cells, functional Na^+^ channels (Na_V_) channels has been detected using the patch clamp technique, and the predominant isoform, the TTX-resistant Na_V_1.5 variant has been reported to be expressed at higher levels compared with the weakly metastatic cell line, MCF-7 [22]. Therefore, abnormal expression of Na_V_1.5 mRNA has been considered an integral component of the metastatic process in human cancer [23],[24]. Likewise, it has been reported that an upregulation of functional Na_V_ channels occurs in MDA-MB-231 cells, particularly the neonatal splice variant of Na_V_1.5 [25]. Based on these findings, we next performed experiments to examine the possible role of the A_2B_ receptor activation on the upregulation of Na_V_1.5 channel expression in MDA-MB-231 cells. To this end, we first compared the mRNA level of the A_2B_ receptor in the MDA-MB-231 cell lines by RT-PCR in presence and absence of Ado. The results of the densitometric analysis showed that the A_2B_ receptor mRNA expression of was ~50% higher in MDA-MB-231 cells treated with Ado (250 μM) than the level of expression in the control cells (Fig 7A). More importantly, the electrophysiological analysis showed that the current density through Na_V_1.5 channels was ~5-fold higher (at 0 mV) in the MDA-MB-231 cell line after 72 h of treatment with 250 μM Ado (Fig 7B and 7C). Next, the specific involvement of Na_V_1.5 channels in upregulating the invasive behavior in MDAMB-231 cells was tested. As can be seen in Fig 8A and 8B, TTX (10 μM) treatment prevented the Ado-induced increase in migration of MDA-MB-231 cells. It is worth clarifying that Na_V_ channels effectively blocked by nM or pM concentrations of TTX are classified as TTX-sensitive, while channels blocked by μM TTX are considered as TTX-resistant [26] as in the case for Na_V_1.5 channels.

## Discussion

Ado has been described as an important regulator in the tumor microenvironment. Under hypoxia or ischemia conditions, the rate of ATP hydrolysis is increased resulting in high levels of extracellular Ado. It is acknowledged that the extracellular levels of Ado are elevated in tumor tissues [12],[15],[27], which is consistent with the hypothesis that Ado is an important factor in growth, development and tumor progression. Interestingly, under hypoxia conditions macrophages show an increased expression of the A_2B_ receptor as a result of increased expression of the HIF-1α that acts as a transcription factor for the receptor [28],[29]. Indeed, there is growing evidence of the A_2B_ receptor involvement in the growth and progression of tumors. Consistent with this, the invasive breast tumor cells MDA-MB 231 have shown high expression levels of the A_2B_ receptor which have gained relevance as a potential diagnostic and prognostic molecular biomarker in cancer [18],[21].

In the present report, we show that Ado may increase cell proliferation in the MDA-MB 231 cells a triple negative breast cancer model. The following are the main findings of our study: i) Ado induces a significant increase in cell proliferation and migration, ii) the MDA-MB 231 cells express the four types of Ado receptors, being the A_2B_ type the most abundantly expressed, iii) the Ado-induced increase in proliferation and migration depends on sustained activation of the A_2B_ receptor, which activates the intracellular adenylate cyclase/cAMP-dependent protein kinase (PKA) signaling pathway, and iv) Ado also increases the functional expression of Na_V_1.5 channels, a potential biomarker in breast cancer.

During tumor growth, the supply of oxygen is restricted to the cells which release ATP to the extracellular medium that result in increased extracellular levels of Ado sufficient to desensitize the A_1_, A_2A_ and A_3_ receptors, but not the Gs protein coupled A_2B_ receptor. This may lead to augmented levels of cAMP and activation of PKA which promote phosphorylation of diverse proteins. A key finding of our study is that the A_2B_ receptor in MDA-MB-231 cells greatly increases proliferation and migration by the activation of the adenylate cyclase/PKA pathway. It is worth mentioning here that this signaling pathway is involved in a myriad of physiological processes including cell growth, proliferation and differentiation.

It should be also noted that A_2B_R is more highly expressed in MDA-MB-231 cancer cells than other adenosine receptors. Interestingly, Mittal *el al*., (2016) have shown that an A_2B_R inhibitor (A2BRi, PSB1115) decreases both experimental and spontaneous metastasis in mouse models of melanoma and triple-negative breast cancer (TNBC). Consistent with this, lentiviral knockdown of the A_2B_R in mouse and human cancer cells reduced metastasis *in vivo* and decreases viability and colony-forming ability, while transiently delaying cell-cycle arrest *in vitro* [30].

On the other hand, the extracellular concentration of Ado depends on the ATP levels and the activity of the ectonucleotidases CD73 and CD39 [31]. Preliminary results of our laboratory suggest that incubation with Ado increases the level of CD73 mRNA in the MDA-MB 231 cells. Based on this, it is reasonable to speculate that the increased levels of CD73 might act as a feedback signal in the proliferation of the MDA-MB 231 cells, by favoring a high concentration of extracellular Ado. Consistent with this idea, it has been reported that CD73 is overexpressed in different types of tumors and cell lines. Interestingly, in breast cancer cells, CD73 overexpression has been associated with invasiveness and drug resistance [32],[33],[34].

Likewise, in MDA-MB-231 cells long-term treatment with Ado increased Nav1.5 channels expression level. Whilst the mechanism(s) by which Ado increases Na_V_1.5 channel transcription is not well understood, there is growing evidence that Na_V_ channel expression can be regulated at different steps, from transcription to post-translation, and that PKA may be involved at different levels in these processes. In particular, PKA seems to be involved in the budding of transporting vesicles from the trans-Golgi network along the exocytic route to the cell membrane. In addition, PKA can regulate acutely the functional expression of Na_V_ channels through phosphorylation of pre-existing channels in the plasma membrane or changing protein levels by altering trafficking and/or mRNA expression. In any case, the elucidation of the mechanism(s) by which Ado increases PKA and Na_V_ channel functional expression is an interesting topic for future studies.

## Supporting Information

S1 Table Oligonucleotides used in this study for RT-PCR experiments. (TIF)

S2 Table Oligonucleotides used in this study for quantitative RT-PCR experiments. (TIF)
