# Supplementary figures and images for: Adenosine Stimulate Proliferation and Migration in Triple Negative Breast Cancer Cells
Source: PLoS One. 2016 Dec 2;11(12):e0167445. doi: 10.1371/journal.pone.0167445 (PMC5135100; doi:10.1371/journal.pone.0167445)

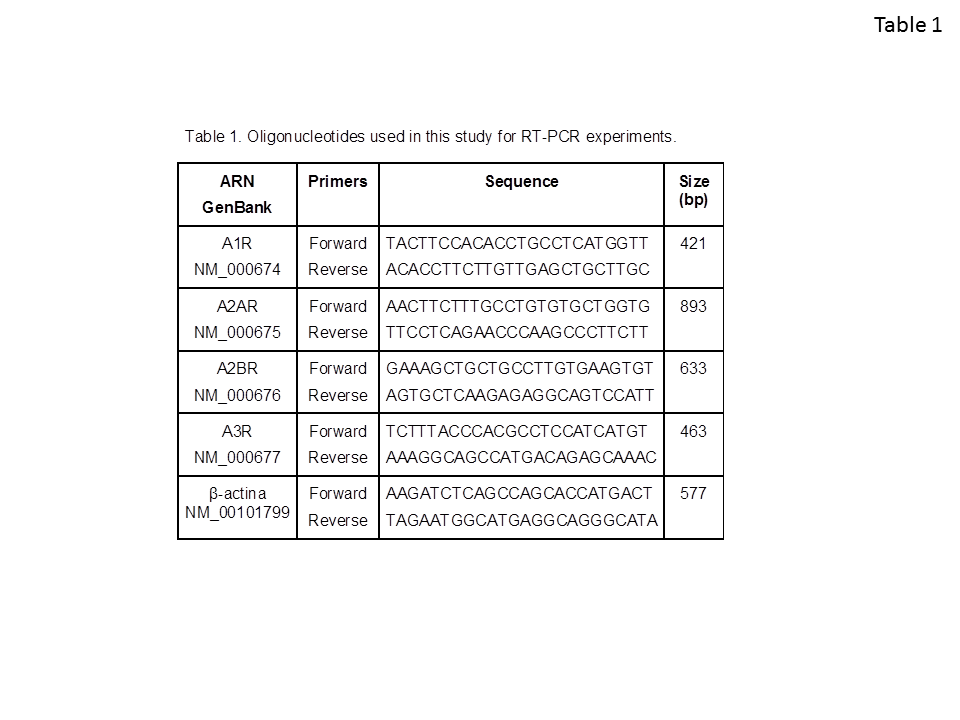

Supplement: S1 Table — (TIF) [file pone.0167445.s001.TIF]

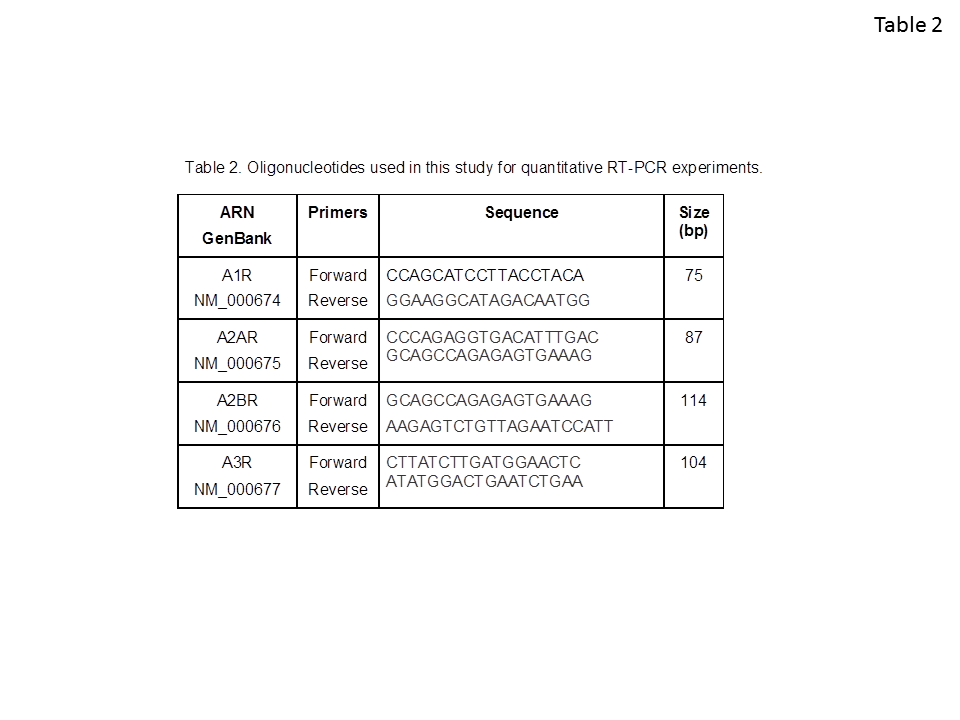

Supplement: S2 Table — (TIF) [file pone.0167445.s002.TIF]
